# A parallel genetic algorithm for single class pattern classification and its application for gene expression profiling in *Streptomyces coelicolor*

**DOI:** 10.1186/1471-2164-8-49

**Published:** 2007-02-13

**Authors:** Cuong C To, Jiri Vohradsky

**Affiliations:** 1 Laboratory of Bioinformatics, Institute of Microbiology, ASCR, Videnska 1083, 142 20 Prague, Czech Republic

## Abstract

**Background:**

Identification of coordinately regulated genes according to the level of their expression during the time course of a process allows for discovering functional relationships among genes involved in the process.

**Results:**

We present a single class classification method for the identification of genes of similar function from a gene expression time series. It is based on a parallel genetic algorithm which is a supervised computer learning method exploiting prior knowledge of gene function to identify unknown genes of similar function from expression data. The algorithm was tested with a set of randomly generated patterns; the results were compared with seven other classification algorithms including support vector machines. The algorithm avoids several problems associated with unsupervised clustering methods, and it shows better performance then the other algorithms. The algorithm was applied to the identification of secondary metabolite gene clusters of the antibiotic-producing eubacterium *Streptomyces coelicolor*. The algorithm also identified pathways associated with transport of the secondary metabolites out of the cell. We used the method for the prediction of the functional role of particular ORFs based on the expression data.

**Conclusion:**

Through analysis of a time series of gene expression, the algorithm identifies pathways which are directly or indirectly associated with genes of interest, and which are active during the time course of the experiment.

## Background

Large scale technologies, such as DNA microarrays or proteomics, provide biologists with the ability to measure the expression levels of thousands of genes in a single experiment. Both methods provide quantitative information about the state of the cell regulatory networks at the moment when the sample was collected. When a particular process is monitored over a longer period of time, samples can be collected in short time intervals. Such an approach generates time series vectors for all detectable genes or proteins, which record activity of the regulatory networks involved in the observed process. Previous experiments suggest that genes sharing similar functions yield similar expression patterns in the microarray or proteomic experiments [1]. Identification of genes that have similar patterns of expression allow for the identification of gene clusters controlled by the same regulator or the identification of processes parallel to the process in which the gene of interest is involved.

So far the identification of specific patterns has been achieved mostly by application of various clustering methods [2-8], where all gene expression time series (profiles) were classified into disjoint groups according to a predefined distance metric. The profiles similar to the profile of the gene of interest are then identified as members of these clusters. Also other approaches based on neural networks [9-11], support vector machines [12-15], or genetic algorithms [16], and others [17] were applied for the classification of transcriptomic and proteomic data. A comprehensive review on the analysis of time series gene expression data was written by Bar-Joseph [18]. Classification methods lead to the identification of genes associated with the cell cycle [19,20], antibiotic biosynthesis [21] or proteins involved in the stress response [22] to name a few.

The classification of genes according to the shapes of their expression profiles has thus become an important issue in the field of systems biology, which allows for the identification of coordinately controlled genes and their associated networks. With the accumulation of knowledge about the role of individual genes, pathways and regulatory networks, it is possible to base the identification of coordinately controlled genes on the previously known roles of particular genes. Those genes then serve as templates for the identification of the other genes which are co-expressed or controlled in the same way as the template gene. In general, such a goal represents a supervised single-class classification problem which was, in the field of transcriptomics, addressed by support vector machine algorithms for the classification of cancer tissue samples [13-15]. Except for the work of Pan et al. [17] that used a rule based system for the gene of interest search in a transcriptomic database, Vohradsky [11] who applied neural networks in the classification of a proteomic time series, and Brown [12] who used SVM, a supervised method based on the previous knowledge of the particular role of a gene or a group of genes has not been reported. Pan et al. identified genes with similar expression profile by using modified Pearson correlation coefficient formula, where a tested gene is compared with a template gene expression profile. Here, we address this problem by utilizing general geometrical concepts and genetic algorithms.

Gene profiles can be viewed as points in multidimensional space with the dimensionality given by the number of measurements. Coordinately controlled genes form a cluster in this space which is more or less separated from the other points. This feature was utilized in cluster analysis which could successfully identify such genes if they were sufficiently separated, otherwise the clustering failed. In this paper, an initial set of genes that are known to share the same function is used as a training set for the suggested algorithm. These points are fitted with a hyperplane which is identified by the genetic algorithm. Other genes are identified as points close to this hyperplane.

Genetic algorithms (GA) are computational methods inspired by Darwinian evolution theory. The variable (or variables) are coded into the vector which is called a chromosome; an initial population of chromosomes is generated randomly. The evolution is performed in an iterative manner where in each step the fitness of chromosomes is evaluated and the population is altered by the operations of crossover, mutation and selection.

Each chromosome can be thought of as a point in the search space of the candidate solutions. The GA processes populations of chromosomes, successively replacing one population by another. The GA most often requires a fitness function that assigns a score (fitness) to each chromosome in the population. In crossover, the operator randomly chooses a locus in the chromosome and exchanges it between two chromosomes to create two offspring. Mutation randomly flips some of the bits in a chromosome. The selection selects chromosomes for reproduction according to their fitness. The fitter the chromosome is, the higher the probability that it will be selected for the next generation. The procedure iterates until the desired fitness is reached or a predefined number of iterations are reached. As the GA is a stochastic process where the initial population is randomly created, and the other operations are also random, usually several runs are done for the same task and the results are evaluated.

In recent years, parallel computing has been applied in evolutionary computing and it has shown not only increased speed, but also the creation of high quality solutions. The parallel scheme can be classified [23] into the following: single population master-salve, multiple-population, fine-grained, and hierarchical hybrids. In this paper, the island model (multiple-population) was applied and we demonstrate here that it can solve the problems better than the sequential GA.

The influence of the parameters of the GA was tested with a set of artificial profiles with different levels of superimposed noise and different dimensions. The performance of the algorithm was demonstrated using the analysis of secondary metabolite gene clusters in the eubacterium *S. coelicolor*. The data were obtained from [24].

## Results and discussion

### Implementation of GA

#### a) Chromosome

Each chromosome was encoded as a fixed-length string of (*n *+ 1) real numbers (value encoding). The first *n *real values represented *u*_*i *_(*i *= 1..*n*); the last real value represented *v*.

#### b) Control parameters of the algorithm

After extensive testing for different sizes of the template vector, we chose the following values of the control parameters, which gave satisfactory results: number of generations = 500, population size = 1000, probability of crossover = 0.9, probability of reproduction = 0.1. Mutation was excluded from the scheme as it substantially increased the computational requirements and did not cause proportional improvement of the algorithm's performance. The choice of parameters was thoroughly discussed in the conference papers of To et al. [25]

#### c) Fitness function

Each chromosome represented a hyperplane, i.e. each chromosome of the initial population was created as a set of (*n *+ 1) real numbers whose values were within the range [-1, 1] satisfying Eq. (5). Eq. (4) was used to calculate the fitness value of each chromosome. The best chromosome, i.e. the chromosome with the smallest value of the fitness function, was selected.

#### d) Parallel scheme

We applied the parallel GA with the following parameters: the ring topology, the migration rate was set to 5% to 10%, migration was executed every 10 generations, and sub-population sizes were 500 for 2 islands and 260 for 4 islands.

### Test using simulated data

The performance of the algorithm was tested by the application of the algorithm to artificial random datasets with different expression profile vector dimensions. The fitness of the population was calculated according to Eq. 4. The template patterns were created randomly and the training set with 20 members from each of the template patterns was created by adding random Gaussian noise to the template profile. The creation of the artificial database (set B) of the profile vectors and the search procedure is summarized in the following steps:

**Step 1**. Random set R of 5000 *n*-dimensional expression profiles was generated.

**Step 2**. Search pattern **x **was defined.

**Step 3**. 100 patterns were created by adding 50% Gaussian noise to the search pattern **x **which generated a set of patterns to be searched (C).

**Step 4**. Set C and R were mixed to form set B (5100 profiles).

**Step 5**. The algorithm was applied to search the set C within the set B.

Performance of the algorithm was tested using two criteria – sensitivity (Se) and specificity (Sp).

Se=TP|C|     (7)
 MathType@MTEF@5@5@+=feaafiart1ev1aaatCvAUfKttLearuWrP9MDH5MBPbIqV92AaeXatLxBI9gBaebbnrfifHhDYfgasaacH8akY=wiFfYdH8Gipec8Eeeu0xXdbba9frFj0=OqFfea0dXdd9vqai=hGuQ8kuc9pgc9s8qqaq=dirpe0xb9q8qiLsFr0=vr0=vr0dc8meaabaqaciaacaGaaeqabaqabeGadaaakeaacqWGtbWucqWGLbqzcqGH9aqpdaWcaaqaaiabdsfaujabdcfaqbqaamaaemaabaGaee4qameacaGLhWUaayjcSdaaaiaaxMaacaWLjaWaaeWaaeaacqaI3aWnaiaawIcacaGLPaaaaaa@3A96@

Sp=|R|−FP|R|     (8)
 MathType@MTEF@5@5@+=feaafiart1ev1aaatCvAUfKttLearuWrP9MDH5MBPbIqV92AaeXatLxBI9gBaebbnrfifHhDYfgasaacH8akY=wiFfYdH8Gipec8Eeeu0xXdbba9frFj0=OqFfea0dXdd9vqai=hGuQ8kuc9pgc9s8qqaq=dirpe0xb9q8qiLsFr0=vr0=vr0dc8meaabaqaciaacaGaaeqabaqabeGadaaakeaacqWGtbWucqWGWbaCcqGH9aqpdaWcaaqaamaaemaabaGaeeOuaifacaGLhWUaayjcSdGaeyOeI0IaemOrayKaemiuaafabaWaaqWaaeaacqqGsbGuaiaawEa7caGLiWoaaaGaaCzcaiaaxMaadaqadaqaaiabiIda4aGaayjkaiaawMcaaaaa@3FEA@

Where

- *TP (true positive)*: the classifier predicts that the pattern is in the set C and the pattern belongs to the set C.

- *FP (false positive)*: the classifier predicts that the pattern is in the set C but the pattern does not belong to the set C.

- |C|: total number patterns in set C (size of set C).

- |R|: total number of other patterns in set R (size of set R).

**Figure 1:**
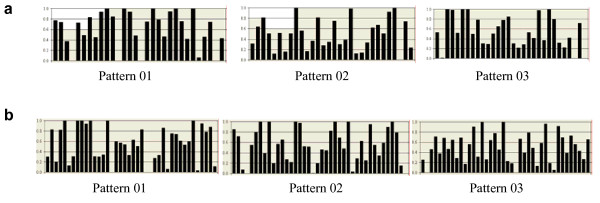
**Three 30 (a), and 40 (b) – dimensional patterns used to test the algorithm**. A training set of 20 patterns from each of the templates was created by adding 50% random Gaussian noise to these templates.

Results are summarized for the profiles of sizes |**x**| = 30 and 40 in Table 1 and 2. Patterns 1–3 mentioned in the Table 1 are shown in Figure 1.

The execution time for one training loop with a training set of size 250 is approximately 3s. For the streptomycetes datasets, which are smaller, the execution time was below 1 s on a Pentium 4 2.4 GHz PC

### Identification of secondary metabolite clusters in *S. coelicolor*

A transcriptomic dataset monitoring 9 time points in the cell cycle of eubacterium *Streptomyces coelicolor *[21] was chosen for evaluation of the algorithm. The database contained temporal expression profiles of 5068 genes forming a 5068 × 9 data matrix. Streptomycetes are known as producers of antibiotics and other secondary metabolites. About 22 gene clusters encoding the secondary metabolites were identified by genome sequence analysis [26]. Identification of such gene clusters from time series experiments serve as a good example of a case where the utilization of a single class classification algorithm is essential.

**Figure 2:**
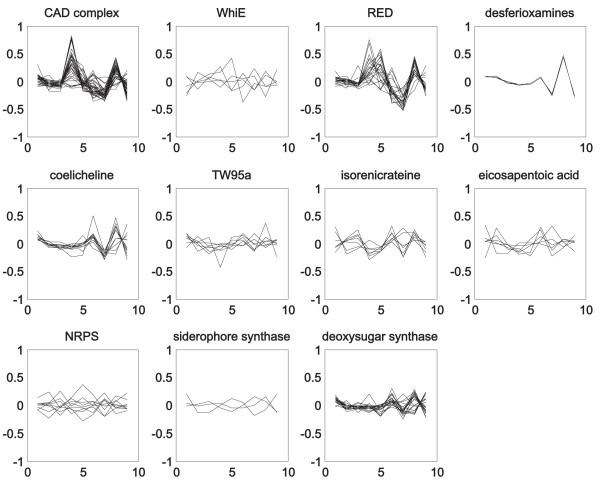
**SVD corrected gene expression profiles of 11 antibiotic and secondary metabolite gene clusters**. The horizontal axis represents time points, whereas the vertical axis represents normalized gene expression level. Expression profiles of RED and coelicheline chromosomal clusters were used in training of the algorithm.

We found that when the data matrix was subjected to singular value decomposition and the first two eigenvectors were subtracted from the matrix, the members of two chromosomal clusters coding for the polyketide antibiotic actinorhodin, the so-called RED complex, and another secondary metabolite cluster specifying the biosynthesis of siderofore coelichelin, exhibited increased similarity (data not shown). Nonetheless, even after such cleaning, the correlation coefficient between the members of the two groups still ranged from -0.92 to 0.67 for the RED gene cluster (-0.99 to 0.83 for the coelicheline cluster) indicating rather high variability in the set which made a search of similar profiles according to the level of correlation or other distance metrics virtually impossible. Not surprisingly, clustering methods failed in the identification of the gene clusters. Individual gene expression profiles of the eleven gene clusters analyzed are shown in Figure 2.

**Figure 3:**
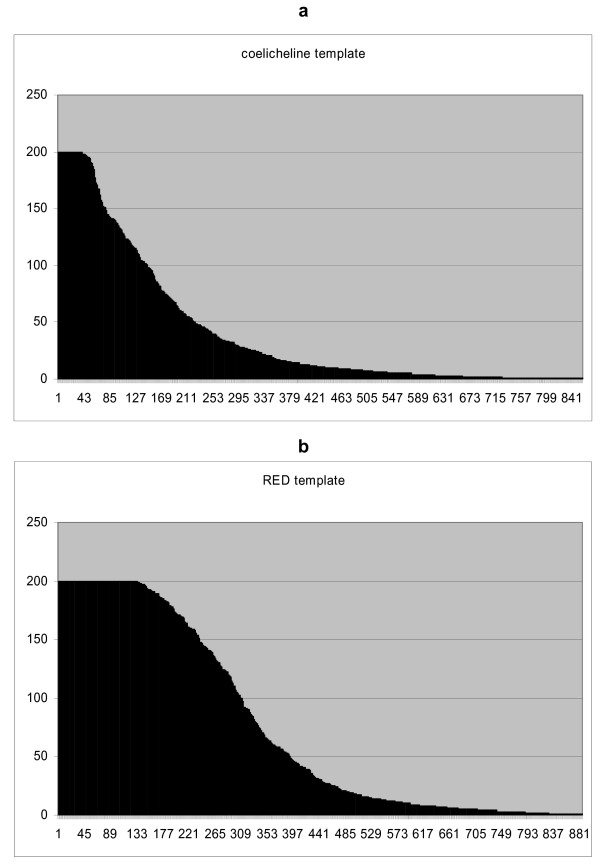
**Scores for identification of expression profiles using (a) coelicheline gene cluster profiles as a training set and (b) RED gene cluster profiles**. The procedure was performed 200 times with random initiation of the chromosome. If a profile was identified in each run it got a score of 200, if it was not found at all it got a score of zero. Vertical axis – scores, horizontal axis – genes sorted according to decreasing scores.

The profiles of these two clusters were used as training sets for GA and through application of the trained algorithms to the whole database of 5068 profiles other genes with kinetics similar to these two templates were identified. The procedure was performed 200 times with random initiation of the chromosomes in each run. All genes identified in each run were scored. This means that if a gene was identified in every run, it got a score of 200. If it was not identified at all, it got a score of zero. Genes were sorted according to their scores and those having scores higher than the threshold were selected. Sorted score values for each gene for both training sets are plotted in Figure 3. Table 3 summarizes the results of the identification of common antibiotic and secondary metabolic clusters with the algorithm trained for the two training sets.

Close inspection of Table 3 shows that genes with an expression profile similar to the RED cluster represented genes of the CAD complex almost exclusively where 28 out of 39 genes were found similar to the RED cluster. This template was found only rarely in other gene clusters. Surprisingly, three genes of the second template, the coelicheline gene cluster, were also found to be similar to the RED template. Comparison of the individual profile shapes of the two gene clusters (see Figure 2) shows that such overlap is possible. Following the logic of the selection, the similar genes of the coelicheline template should be found in the RED gene cluster. Indeed, two were found (see Table 3).

The coelicheline template profiles were also found in two gene clusters – deoxysugar synthase and desferioxamines. Other gene clusters did not show a gene cluster specific profile shape (see Figure 2) and the two template profiles were not found among them, indicating a different control.

Other genes identified as similar to the two template gene clusters are listed in Tables 1 and 2 [see additional file 1] together with their function in the cell metabolism, as defined by The Sanger Institute that sequenced the organism [27]. The threshold value (see above) for inclusion of a gene to the search list was arbitrarily set to 200 for RED and to 100 for coelicheline clusters respectively, generating output sets of similar sizes. It can be expected that these genes are directly or indirectly associated with the secondary metabolite production.

All together 135 genes were identified as similar to the RED gene cluster. Out of these 89 were annotated, 9 were not classified, most of them (46 genes, 58%), fell into a group "Secondary metabolism" as expected. Second largest group (14 genes, 18%) was associated with the cell envelope. The remaining genes were associated with the metabolism of small molecules, regulation and transport/binding processes.

**Figure 4:**
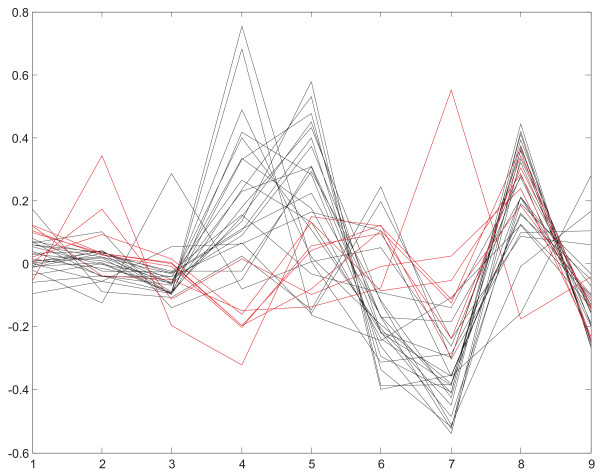
Expression profiles of RED gene cluster (black) and ECR genes identified by Huang et al. [21] (red).

Huang et al. [21] using the same dataset identified, using GABRIEL software [17], so called ECR genes i.e genes correlated with RED gene expression cluster. They were (SCO6423, SCO6421, SCO6422, SCO4332, SCO2716, SCO2518, SCO2517, SCO2519). Our algorithm identified none of them. In order to find the reason for such difference, we plotted the expression profiles of the RED cluster genes together with the expression profiles of the genes found by Huang et al. (plotted in red in Figure 4). Figure 4 shows that the ECR genes follow the RED gene profiles only in the early and late phases therefore our algorithm could not identify them.

Using the coelicheline gene cluster as a training set, a total of 97 genes were identified and annotated, of which 20 were not classified or were classified as "others". Out of the remaining 77 genes, 24 (31%) were classified as "secondary metabolism" genes. The second largest group was formed with 20 genes (26%) associated with membrane function and building, similar to the previous case. As the secondary metabolites have to be exported from the cell, most of the gene clusters coding for their pathways include also genes coding for membrane proteins. Therefore identification of genes involved in cell wall function is in good agreement with previous findings.

The remaining individual genes similar to the templates were associated with transport, regulation and other different particular functions (Table 2 [see additional file 1]). The association of the genes of the transport mechanism can also be expected as the secondary metabolites have to be exported out of the cell. This diversity can be caused by true similarity of the regulation of different processes by different regulators without any connection among the corresponding networks. In individual cases, the similarity to the given template can also be caused by experimental inaccuracies or noise which are both known to be rather high in microarray experiments. Such cases have to be assessed individually and where appropriately verified by independent methods such as qPCR. More detailed biological interpretation of the results is out of scope of this paper.

## Conclusion

The presented algorithm falls into a class of single-class problems which has gained increasing attention in bioinformatics over the last few years (see e.g. [28]). For the single-class problem, we want a given dataset to estimate a subset such that the probability that a test point drawn from the dataset lying outside of the subset equals some a priori specified value between 0 and 1. The goal is to find a function which is positive on the desired subset and zero or negative on the complement. In this paper, we provide evidence that the identification of a hyperplane using a GA is quite suitable for this task, allowing for the identification of user-defined gene expression time series templates in a large set of profiles.

The demand for the identification of user-defined templates of gene expression profiles increases with the availability of large-scale gene expression data when microarray or proteomic experiments cover whole cell cycles or other time evolving processes. The typical genome size, and thus the number of genes immobilized on a microarray, exceed tens of thousands. The number of time series in an experiment also exceeds this number. To search through such a database is a nontrivial task. With the increasing knowledge about the regulation of gene expression such datasets can be approached with existing knowledge of the system. Therefore the initial classification of the profiles into disjoint clusters can now be replaced by targeted searches for genes which have kinetics similar to the gene with already known function. Such genes can be under the control of the same promoter, or they can participate in the same regulatory process, or the processes in which they are involved can be parallel to the process represented by the genes of the training set. Their identification is essential for elucidating both their control and their role in the studied process. In such cases approaching the problem as a single-class problem is appropriate.

Here we demonstrate that application of the algorithm presented here, trained on one known gene cluster, can be used in the search of other gene clusters of a similar type even when the variability of the correlation between the members of the training set is quite high (see Results and Discussion). Such variability makes identification based on correlation or other distance metrics impossible. Compared to other classifiers, our algorithm displays better accuracy.

Besides the identification of known gene clusters in gene expression data of a model organism *S. coelicolor*, which confirm the capability of the method to identify known patterns, new genes similar to the template gene clusters were identified. As expected, most of them fall into a group of genes of secondary metabolism located on different parts of the chromosome. Their function can be deduced from sequence similarity but it can not be proven that the genes are controlled in the same way as the known gene clusters. The method presented here provides such evidence. In contrast with the genome annotation methods which can identify potential gene clusters, this method is capable of identifying pathways associated with the searched function which are active. The algorithms not only identify the genes of the main pathway, in this case antibiotic biosynthesis, but also genes which are associated with it. Here, the genes are those of the transport mechanism or cell wall function. Therefore this method allows the identification of whole active networks participating in the expression of genes of interest. Results showed that the method presented here is more powerful in identification of associated pathways than other pattern recognition algorithms and/or frequently used correlation analysis. Also the application of this algorithm to a less studied organism can lead to the identification of unknown gene clusters and associated pathways.

In general the disadvantage of evolutionary methods is the high computational requirement. We bypassed this problem by the introduction of a parallel computational scheme which greatly increases the speed of computation. Moreover, the parallel scheme suggested here improves the performance of the algorithm. Nowadays, multiprocessor machines or computer clusters are easily available and the parallel programming is no longer only in the domain of large computers. Therefore, implementation of the parallel algorithm is feasible. In closing, the algorithm presented here is very fast, and the execution of one run is counted in seconds or fractions of seconds on an ordinary PC.

## Methods

The goal of the presented algorithm is the supervised identification of expression profiles. In this paper, the term 'expression profile' or just 'a profile' means a time series vector of individual mRNA amounts measured during a particular process **x **= [*x*_1_, *x*_2_,..., *x*_*n*_]^T^, where *n *is the number of measurements. In principle, the profile can be any vector formed by a series of measurements. Initially, a small set of gene profiles that are known to be coordinately controlled is selected. This set is called a training set. During the training process, the genetic algorithm identifies a hyperplane which has a minimum distance from the vectors of the training set. Then all profiles from the database, which have the same or smaller distance from the hyperplane than the training set, are selected. Thus, these profiles have the desired similarity to the template profile.

### Concepts from geometry

#### Hyperplane

Let *u*_1_, *u*_2_, ..., *u*_*n*_, *v *∈ R, where at least one of the *u*_*i *_is nonzero. The set of all points **x **= [*x*_1_, *x*_2_,..., *x*_*n*_]^T ^that satisfy the linear equation

∑i=1nuixi=v     (1)
 MathType@MTEF@5@5@+=feaafiart1ev1aaatCvAUfKttLearuWrP9MDH5MBPbIqV92AaeXatLxBI9gBaebbnrfifHhDYfgasaacH8akY=wiFfYdH8Gipec8Eeeu0xXdbba9frFj0=OqFfea0dXdd9vqai=hGuQ8kuc9pgc9s8qqaq=dirpe0xb9q8qiLsFr0=vr0=vr0dc8meaabaqaciaacaGaaeqabaqabeGadaaakeaadaaeWbqaaiabdwha1naaBaaaleaacqWGPbqAaeqaaOGaemiEaG3aaSbaaSqaaiabdMgaPbqabaGccqGH9aqpcqWG2bGDaSqaaiabdMgaPjabg2da9iabigdaXaqaaiabd6gaUbqdcqGHris5aOGaaCzcaiaaxMaadaqadaqaaiabigdaXaGaayjkaiaawMcaaaaa@3FF4@

is called a hyperplane in the space R^n^. We may describe the hyperplane by

{**x **∈ R^*n *^: **u**^*T *^**x **= *v*}   (2)

#### Distance from the point to the hyperplane

Given a point **a **= [*a*_1_, *a*_2_, ..., *a*_*n*_]^T ^and a hyperplane *H *= {**x **∈ R^*n *^: **u**^*T *^**x **= *v*}, the distance from the point to the hyperplane is defined as

d(a,H)=|uTa−v|∑i=1nui2     (3)
 MathType@MTEF@5@5@+=feaafiart1ev1aaatCvAUfKttLearuWrP9MDH5MBPbIqV92AaeXatLxBI9gBaebbnrfifHhDYfgasaacH8akY=wiFfYdH8Gipec8Eeeu0xXdbba9frFj0=OqFfea0dXdd9vqai=hGuQ8kuc9pgc9s8qqaq=dirpe0xb9q8qiLsFr0=vr0=vr0dc8meaabaqaciaacaGaaeqabaqabeGadaaakeaacqWGKbazcqGGOaakieqacqWFHbqycqGGSaalcqWGibascqGGPaqkcqGH9aqpdaWcaaqaamaaemaabaGae8xDau3aaWbaaSqabeaacqWGubavaaGccqWFHbqycqGHsislcqWG2bGDaiaawEa7caGLiWoaaeaadaGcaaqaamaaqahabaGaemyDau3aa0baaSqaaiabdMgaPbqaaiabikdaYaaaaeaacqWGPbqAcqGH9aqpcqaIXaqmaeaacqWGUbGBa0GaeyyeIuoaaSqabaaaaOGaaCzcaiaaxMaadaqadaqaaiabiodaZaGaayjkaiaawMcaaaaa@4C71@

#### Algorithm

Let define a training set as a set of similar pattern *TS *= {**x**_*i *_∈ R^n^, *i *= 1..*m*}. As the gene expression profile similarity does not depend on the amplitude, all profiles are normalized to the interval <0,1> **(x **⇐ **x**/max **(x))**. The main idea of the algorithm is to find a hyperplane *H *that can contain all points of the training set. In other words, find a hyperplane *H *that minimizes the total distance from all points in the training set to the hyperplane *H*. Therefore it is necessary to find

**z **= [*u*_1_, *u*_2_, ..., *u*_*n*_, *v*]^T ^which minimizes

f(z)=∑k=1md(xk,H)=∑k=1m|uTxk−v|∑i=1nui2     (4)
 MathType@MTEF@5@5@+=feaafiart1ev1aaatCvAUfKttLearuWrP9MDH5MBPbIqV92AaeXatLxBI9gBaebbnrfifHhDYfgasaacH8akY=wiFfYdH8Gipec8Eeeu0xXdbba9frFj0=OqFfea0dXdd9vqai=hGuQ8kuc9pgc9s8qqaq=dirpe0xb9q8qiLsFr0=vr0=vr0dc8meaabaqaciaacaGaaeqabaqabeGadaaakeaacqWGMbGzcqGGOaakieqacqWF6bGEcqGGPaqkcqGH9aqpdaaeWbqaaiabdsgaKjabcIcaOiab=Hha4naaBaaaleaacqWGRbWAaeqaaOGaeiilaWIaemisaGKaeiykaKIaeyypa0ZaaabCaeaadaWcaaqaamaaemaabaGae8xDau3aaWbaaSqabeaacqWGubavaaGccqWF4baEdaWgaaWcbaGaem4AaSgabeaakiabgkHiTiabdAha2bGaay5bSlaawIa7aaqaamaakaaabaWaaabCaeaacqWG1bqDdaqhaaWcbaGaemyAaKgabaGaeGOmaidaaaqaaiabdMgaPjabg2da9iabigdaXaqaaiabd6gaUbqdcqGHris5aaWcbeaaaaaabaGaem4AaSMaeyypa0JaeGymaedabaGaemyBa0ganiabggHiLdaaleaacqWGRbWAcqGH9aqpcqaIXaqmaeaacqWGTbqBa0GaeyyeIuoakiaaxMaacaWLjaWaaeWaaeaacqaI0aanaiaawIcacaGLPaaaaaa@6368@

Subjected to:

{−1≤ui≤1,i=1..n, and ∃ui≠0−1≤v≤1     (5)
 MathType@MTEF@5@5@+=feaafiart1ev1aaatCvAUfKttLearuWrP9MDH5MBPbIqV92AaeXatLxBI9gBaebbnrfifHhDYfgasaacH8akY=wiFfYdH8Gipec8Eeeu0xXdbba9frFj0=OqFfea0dXdd9vqai=hGuQ8kuc9pgc9s8qqaq=dirpe0xb9q8qiLsFr0=vr0=vr0dc8meaabaqaciaacaGaaeqabaqabeGadaaakeaadaGabeqaauaabeqaceaaaeaacqGHsislcqaIXaqmcqGHKjYOcqWG1bqDdaWgaaWcbaGaemyAaKgabeaakiabgsMiJkabigdaXiabcYcaSiabdMgaPjabg2da9iabigdaXiabc6caUiabc6caUiabd6gaUjabcYcaSiabbccaGiabbggaHjabb6gaUjabbsgaKjabbccaGiabgoGiKiabdwha1naaBaaaleaacqWGPbqAaeqaaOGaeyiyIKRaeGimaadabaGaeyOeI0IaeGymaeJaeyizImQaemODayNaeyizImQaeGymaedaaaGaay5EaaGaaCzcaiaaxMaadaqadaqaaiabiwda1aGaayjkaiaawMcaaaaa@56D5@

To solve the above nonlinear programming problem, we used a genetic algorithm.

### Genetic algorithm

The initial population of chromosomes is created randomly. Here we use value encoding of the variables to the chromosome where each chromosome contains real numbers representing the vector **z**. Using the crossover and reproduction operations a new generation is created. The fitness of the chromosomes is evaluated using the fitness function defined by Eq. 4. The whole scheme can be summarized into the following steps:

(1) Generate an initial population of random chromosomes.

(2) Iteratively perform the following sub-steps until the termination criterion is satisfied:

(a) Calculate the fitness function of each chromosome in the population.

(b) Create a new population of chromosomes by applying the crossover and reproduction operations with probability given by the fitness of individual chromosomes.

(3) The best chromosome, i.e. the chromosome with the lowest value of the fitness function appearing in any generation, is selected as the result of the GA. This represents the best approximation of the solution.

Each chromosome of the population is a candidate solution of the problem. The termination criterion can be either a maximum number of generations which are allowed to be generated or a desired minimal value of the fitness function.

The training process was performed 100 times for randomly set initial values of parameters and a parameter set giving the best fitness level was selected.

### Implementation for large templates

With increasing profile vector length, the demands for the memory and computer time increase. Additionally the probability of obtaining better fitness by reproduction and crossover decreases with the length of the template. Therefore to get the desired accuracy it is necessary to compute more generations and thus to increase the processing time. We have adapted the algorithm to improve the computational efficiency in the following way:

Let assume that the dimension of vector **x **is *r*. We split the problem *rs *of classification of pattern **x **into *k *disjunctive sub problems *rs*_*i *_(*i = 1, k*) whose dimensions are *r*_*i *_and ∑i=1kri=r
 MathType@MTEF@5@5@+=feaafiart1ev1aaatCvAUfKttLearuWrP9MDH5MBPbIqV92AaeXatLxBI9gBaebbnrfifHhDYfgasaacH8akY=wiFfYdH8Gipec8Eeeu0xXdbba9frFj0=OqFfea0dXdd9vqai=hGuQ8kuc9pgc9s8qqaq=dirpe0xb9q8qiLsFr0=vr0=vr0dc8meaabaqaciaacaGaaeqabaqabeGadaaakeaadaaeWbqaaiabdkhaYnaaBaaaleaacqqGPbqAaeqaaOGaeyypa0JaemOCaihaleaacqWGPbqAcqGH9aqpcqaIXaqmaeaacqqGRbWAa0GaeyyeIuoaaaa@390B@.

The results for *k *sub-patterns are then combined according to Eq. (6) to get the result for the original pattern:

rs=∩i=1krsi     (6)
 MathType@MTEF@5@5@+=feaafiart1ev1aaatCvAUfKttLearuWrP9MDH5MBPbIqV92AaeXatLxBI9gBaebbnrfifHhDYfgasaacH8akY=wiFfYdH8Gipec8Eeeu0xXdbba9frFj0=OqFfea0dXdd9vqai=hGuQ8kuc9pgc9s8qqaq=dirpe0xb9q8qiLsFr0=vr0=vr0dc8meaabaqaciaacaGaaeqabaqabeGadaaakeaacqWGYbGCcqWGZbWCcqGH9aqpdaafWbqaaiabdkhaYjabdohaZnaaBaaaleaacqWGPbqAaeqaaaqaaiabdMgaPjabg2da9iabigdaXaqaaiabdUgaRbqdcqWIPissaOGaaCzcaiaaxMaadaqadaqaaiabiAda2aGaayjkaiaawMcaaaaa@3F2E@

where:

- rs_i _is the resulting set of the problem of the *i*-th sub pattern and rs is the resulting set of the original pattern_._

In principle, it means searching the best hyperplanes for all subintervals of dimension *r*._*i*. _Their combination is then used to search the profiles similar to the desired pattern from the database.

### Parallel scheme

Among the four major types of parallel GAs mentioned in the introduction, the island model is rather complicated but shows very good performance [23]. In the island model, the population is partitioned into sub-populations. Each sub-population, called an island, is assigned to one processor and runs independently. After a predefined number of generations, islands exchange part of the chromosomes with each other, this process is called migration. This model has been applied to many problems [29-31] and has shown that not only does it increases the performance of the algorithm, but it also gives better results than the sequential algorithm. In order to use the island model, we had to determine its parameters i.e. topology, migration rate, migration frequency, and sub-population size.

The topologies which can be considered are the following: grid, ring, and random. de Vega [31] introduced a random topology and compared it with the grid and ring topologies. He concluded that if all other parameters are kept fixed, there is no significant difference among the topologies. By testing four model problems, he showed that the best migration rate is between 5% to 10% of the sub-population of chromosomes and the best convergence results appear when about 10% from each sub-population was exchanged every 5–10 generations.

Calegari [30] showed that the performance of the algorithm grows with an increasing number of islands implemented and a decreasing number of chromosomes in each single island.

### Classification

After the hyperplane *H i*s identified, the distances of all points of the training set to the resulting hyperplane *H *is calculated. Then the maximum distances *f*_max_(**x**_i_, i = 1..m) of the points of the training set from the hyperplane are calculated. If the distance *d*(**a**, *H*) of the tested point **a **from the resulting hyperplane *H *lies between the hyperplane and *f*_max_, then the tested point **a **is defined as similar to the training set *TS*. A more sophisticated criterion based on the analysis of distribution of the distances of the points of the training set from the hyperplane can be implemented. Instead we chose the simplest criterion mentioned above which despite its simplicity gives satisfactory results. The procedure gradually tests all profiles in the database and selects those satisfying this criterion. Selectivity of the procedure is given by the mutual similarity of the profiles in the training set. The more similar the profiles in the training set are, the more selective the algorithm is.

The training set can be created from a selection of profiles in the database or by defining one profile where the training set is created by adding random noise to this profile.

The algorithm presumes that the expressions of the individual genes of the database were measured at the same time points. If the measurement time points differ for different genes, it is necessary prior to the analysis to align the time scales of the different measurements. The way to align such experiments was thoroughly discussed in the work of Bar-Joseph [18].

### User interaction

When the classification of patterns has to incorporate particular knowledge which is either difficult to cast into an algorithm or which is intuitive or difficult to define, user interaction with the pattern search algorithm is implemented into the process. We wanted to incorporate an undefined expert knowledge by introducing the user's decision to each iteration step during the training process. The intervention of the user is based on a modification of the training set after each run. From the point of view of the GA, the task is further specified between two consecutive runs by the user through the addition of new profiles or the removal of selected profiles from the training set. The whole procedure involving the user interaction is summarized below:

**Step 1**. The user inputs the patterns in the training set. The patterns can be selected either from a database of patterns or alternatively, the user defines one template profile and the training set is created by adding random noise to this template.

**Step 2**. GA finds the hyperplane.

**Step 3**. Max and min distance (*f*_max_(**x**_i_, i = 1..m) and *f*_min_(**x**_i_, i = 1..m)) of the members of the training set from the hyperplane are identified.

**Step 4**. The algorithm searches through the database and the patterns whose distance from the hyperplane is within <*f*_min_(**x**_i_, i = 1..m), *f*_max_(**x**_i_, i = 1..m) > are selected.

**Step 5**. The selected patterns are checked by the user. The misclassified (false positive) ones are removed from the set. The set becomes a new training set.

**Step 6**. If new correct patterns are found go to step 2, or else terminate.

**Figure 5:**
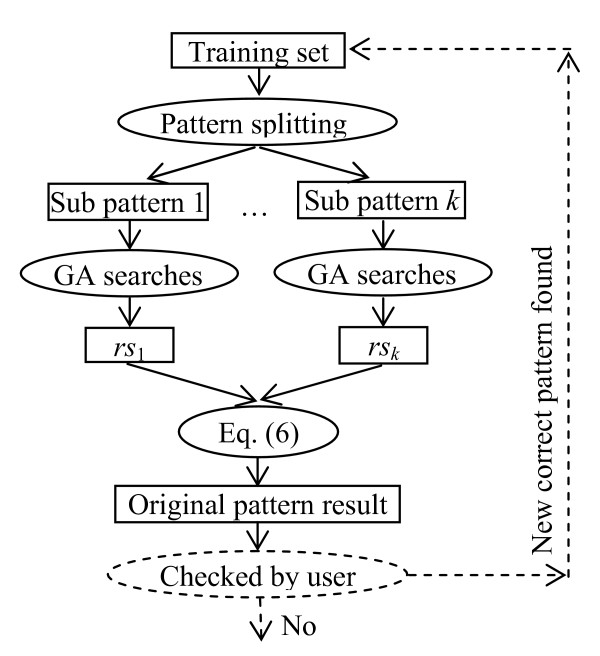
Overall scheme of the algorithm.

The overall scheme including pattern splitting is given in Figure 5.

## Authors' contributions

CT designed the hyperplane concept, wrote the code and carried out the computations. JV conceived of the study, and participated in its design and coordination, drafted the manuscript, made the biological interpretation of the results. All authors read and approved the final manuscript.

## Supplementary Material

Additional file 1 **Supplementary tables**. Table 1. List of genes with expression profiles similar to the coelicheline gene cluster. Table 2. List of genes with expression profiles similar to the RED gene cluster. Click here for file

## Figures and Tables

**Table 1:** Comparison of classification performance of the parallel and sequential GA with other classification algorithms |x| = 30.

Pattern	Sequent GA		Parallel GA (2 islands)		Parallel GA (4 islands)		Binary SVM		Single SVM		LogitBoost		LR		LDA		LS	
	
	*Se*	*Sp*	*Se*	*Sp*	*Se*	*Sp*	*Se*	*Sp*	*Se*	*Sp*	*Se*	*Sp*	*Se*	*Sp*	*Se*	*Sp*	*Se*	*Sp*
1	1	0.9874	1	0.988	1	0.9902	1	0.987	0.95	0.8576	1	0.837	1	0.8168	1	0.8664	0.9875	0.8666
2	1	0.9764	1	0.9772	1	0.9784	1	0.9766	0.95	0.6488	0.9875	0.8296	1	0.2624	0.9375	0.8766	0.9	0.8768
3	1	0.9644	1	0.966	1	0.9708	1	0.9682	0.8375	0.9378	0.9625	0.9018	0.9625	0.8876	0.95	0.9016	0.9375	0.9018

Sequential GA, parallel GA (2 and 4 islands mode) and support vector machines (SVM binary and single), logitBoost, linear discriminant analysis (LDA), logistic regression (LR), and linear least squares regression (LS) for three sets of template vectors of different dimensions were tested. Se and Sp are defined in Eq. 7 and 8.

**Table 2:** Comparison of classification performance of the parallel and sequential GA with other classification algorithms |x| = 40.

Pattern	Sequent GA		Parallel GA (2 islands)		Parallel GA (4 islands)		Binary SVM		Single SVM		LogitBoost		LR		LDA		LS	
	
	*Se*	*Sp*	*Se*	*Sp*	*Se*	*Sp*	*Se*	*Sp*	*Se*	*Sp*	*Se*	*Sp*	*Se*	*Sp*	*Se*	*Sp*	*Se*	*Sp*
01	1	0.9786	1	0.9862	1	0.9922	1	0.9884	0.925	0.959	1	0.4906	1	0.6602	1	0.2076	0.9625	0.6234
02	1	0.9836	1	0.9858	1	0.9866	1	0.9556	0.8375	0.9918	0.9875	0.6846	1	0.326	1	0.2152	0.975	0.8174
03	1	0.9928	1	0.995	1	0.9972	1	0.9844	0.8875	0.8452	-	-	1	0.7042	1	0.3732	0.3375	0.3866

Sequential GA, parallel GA (2 and 4 islands mode) and support vector machines (SVM binary and single), logitBoost, linear discriminant analysis (LDA), logistic regression (LR), and linear least squares regression (LS) for three sets of template vectors of different dimensions were tested. Se and Sp are defined in Eq. 7 and 8.

**Table 3:** Results of the search of eleven secondary metabolic gene clusters of S. coelicolor using the GA algorithm trained with kinetic profiles of RED antibiotic and coelicheline gene clusters (in bold).

cluster	SCO beginning	SCO end	n	RED template	%	Coelicheline template	%
CAD complex	3210	3249	39	28	72	2	5
whiE	5327	5350	5	0	0	0	0
**RED**	5877	5898	22	NA	NA	2	9
desferioxamines	2782	2785	4	1	25	3	75
**coelicheline**	489	499	11	3	27	NA	NA
TW95a	5314	5320	7	1	14	0	0
isorenicratein	185	191	7	0	0	0	0
eicosapentoic acid	124	129	6	0	0	0	0
NRPS	6429	6438	9	0	0	0	0
siderophore synthase	5799	5801	3	0	0	0	0
deoxysugar synthase	381	401	21	1	5	12	57

Gene clusters for act, coelibactine, tetrahydroxy naftalene, type I polyketide, chalcone synthase, sesquiterpene, type III fatty acid synthase were not present in the chip data matrix. Geosmine and butyrolactone represented only one gene and were therefore excluded from evaluation. SCO beginning and end represent beginning and end of the gene cluster on the chromosome, where n = number of genes in a gene cluster, RED template refers to the number of genes of a gene cluster identified using RED gene cluster as a training set, and coelicheline template refers to the number of genes of a gene cluster identified using the coelicheline gene cluster as a training set.
