# Supplementary material for: A parallel genetic algorithm for single class pattern classification and its application for gene expression profiling in Streptomyces coelicolor
Source: BMC Genomics. 2007 Feb 13;8:49. doi: 10.1186/1471-2164-8-49 (PMC1804277; doi:10.1186/1471-2164-8-49)
Supplement: Additional file 1 — Supplementary tables. Table 1. List of genes with expression profiles similar to the coelicheline gene cluster. Table 2. List of genes with expression profiles similar to the RED gene cluster. [file 1471-2164-8-49-S1.pdf]

A Parallel Genetic Algorithm for Single Class Pattern Classification and its Application for Gene Expression Profiling in *Streptomyces coelicolor*

Cuong To and Jiri Vohradsky*

Supplementary tables.

Table 1. List of genes with expression profiles similar to the coelicheline gene cluster

chipID	Gene	SCO	sangerID	sanger function	sanger function group
1103	SCF34.03C	SCO0484	142	1.4.2 Detoxification	1.4.2 Detoxification
637	SCE46.17	SCO3460	142		
3529	SCF43A.15	SCO0825	150	1.5.0 Transport/binding proteins	1.5.0 Transport/binding proteins
2676	SCC22.10	SCO1928	150		
2936	SCC75A.18	SCO2272	150		
2794	SC6A9.09C	SCO5658	150		
4592	SC9G1.02	SCO6212	150		
1034	RAMA	SCO6683	150		
2909	SCF11.15	SCO0535	153		
1641	SCF51.08C	SCO0409	181	**1.8.1 Differentiation/sporulation**	
1417	SCE65.32C	SCO3496	213	2.1.3 Degradation of polysaccharides	
76	SCL6.08C	SCO1451	214	2.1.4 Degradation of proteins, peptides, glycoproteins	
3410	SCI35.35C	SCO1613	318	3.1.8 Glutamine	3.0.0 Metabolism of small molecules
4395	SC7A8.33C	SCO2494	334	3.3.4 Gluconeogenesis	
1756	SCAH10.13	SCO6248	345	3.4.0 Degradation of small molecules	
3430	NUON	SCO4575	353	3.5.0 Energy metabolism, carbon	
273	SC1A6.04	SCO6415	372	3.7.2 Pyrimidine ribonucleotide biosynthesis	
3747	SCF62.12	SCO0386	380	3.8.0 Secondary metabolism	3.8.0 Secondary metabolism
3210	SCF62.13	SCO0387	380		
2704	SCF62.14	SCO0388	380		
2217	SCF62.15	SCO0389	380		
659	SCF62.18	SCO0392	380		
2991	SCF62.20	SCO0394	380		
2488	SCF62.21	SCO0395	380		
459	SCF62.25	SCO0399	380		
2430	SCF34.08C	SCO0489	380		
2304	SCF34.09	SCO0490	380		
4041	SCF34.10C	SCO0491	380		
508	SCF34.12C	SCO0493	380		
3809	SCF34.13C	SCO0494	380		
2058	SCF34.14C	SCO0495	380		
295	SCF34.15C	SCO0496	380		
1828	SCF34.17C	SCO0498	380		
1050	SCF34.18	SCO0499	380		
374	SCE8.09	SCO3216	380		
710	SCE8.16C	SCO3223	380		
4037	SCE8.17C	SCO3224	380		
4133	SCE29.16C	SCO3247	381		
1651	REDV	SCO5882	381		
2269	SCF34.11C	SCO0492	382		

1086	CDAR	SCO3217	382		
4511	SCM2.12C	SCO0859	416	4.1.6 Gram +ve membrane	4.0.0 Cell envelope
2514	SCM2.16C	SCO0863	416		
4377	SC6D7.07	SCO1432	416		
2946	SC3A3.01	SCO2023	416		
496	SCC75A.16C	SCO2270	416		
4521	SC7A8.31	SCO2492	416		
2551	SCC121.23C	SCO2520	416		
3205	SCE20.31C	SCO2857	416		
744	SCE15.05	SCO3288	416		
979	SC1C2.11	SCO5530	416		
1923	SC4C6.18	SCO6708	416		
4341	SC5H1.32	SCO7260	416		
4843	SC5H1.31	SCO7261	416		
1488	SCF62.04C	SCO0378	417	4.1.7 Gram +ve exported/lipoprotein	
2933	SCF43.09	SCO0798	417		
2359	SCI11.14C	SCO1725	417		
684	SCC77.17C	SCO2550	417		
717	SC8E4A.20C	SCO2650	417		
3591	SCE36.08	SCO3441	417		
2982	SC7F9.23C	SCO6871	417		
1992	SCH35.10	SCO3714	514	5.1.4 Transposon/insertion element-related functions	5.0.0 Extrachromosomal
4243	SC3C8.15	SCO6396	514		
3439	SCF11.31C	SCO0551	611	6.1.1 Sensor kinase	6.0.0 Regulation
3525	SC1A6.13C	SCO6424	611		
3797	SCJ1.08	SCO0159	621	6.2.1 sigma factor	
2522	SCF91.32	SCO0672	623	6.2.3 anti sigma factor antagonist	
4140	RSTP	SCO3943	635	6.3.5 LacI	
3096	SC4B5.05C	SCO6555	636	6.3.6 LysR	
2376	SCF81.04C	SCO0745	638	6.3.8 TetR	
5014	SC1C2.13	SCO5532	638		
2620	SC5A7.31	SCO6681	641	6.4.1 Serine/threonine	
618	SCF41.29C	SCO0370	650	6.5.0 Others	
1400	SCL2.31	SCO1541	650		
222	SCI8.24C	SCO1839	650		
1946	SCE19A.24	SCO2924	650		
5052	SCE19A.35C	SCO2935	650		
804	SCH66.08C	SCO3587	650		
3061	SCF91.05C.	SCO0645	700	7.0.0 Not classified (included putative assignments)	
2074	SCF43A.31	SCO0841	700		
1865	SCI35.33C	SCO1611	700		
117	SCI35.34C	SCO1612	700		
4618	SCI51.06C	SCO1766	700		
2827	SCI51.07C	SCO1767	700		
2175	SCC54.08C	SCO1948	700		
350	SCC121.29	SCO2526	700		
4405	SCE65.13C	SCO3477	700		
4668	SCE2.15	SCO3534	700		
600	SC9B5.08	SCO6441	700		
100	SC9B5.09	SCO6442	700		
622	SC7F9.33C	SCO6881	700		
1243	SC5H1.38	SCO7254	700		
965	SCI7.13C	SCO1895	3113		

1618	SCH69.12	SCO3842	3115		
2001	SCF43A.18	SCO0828	3313		
594	SCJ21.13	SCO0132	6311		
3085	SCE39.25C	SCO3275	6312		

Table 2. List of genes with expression profiles similar to the RED gene cluster

chipID	Gene	SCO	sangerID	sanger function	sanger function group
1034	RAMA	SCO6683	150	1.5.0 Transport/binding proteins	1.5.0 Transport/binding proteins
2794	SC6A9.09C	SCO5658	150		
2936	SCC75A.18	SCO2272	150		
3840	SC7B7.04	SCO6007	150		
3048	SCG11A.18	SCO1187	213	2.1.3 Degradation of polysaccharides	2.1.0 Macromolecule degradation
4917	SC3A7.15	SCO6347	213		
3410	SCI35.35C	SCO1613	318	3.1.8 Glutamine	3.0.0 Metabolism of small molecules
4793	SCJ12.20	SCO0208	334	3.3.4 Gluconeogenesis	
4682	SC5G9.21	SCO0312	344	3.4.4 Fatty acids	
3430	NUON	SCO4575	353	3.5.3 Electron transport	
3467	SCM10.12C	SCO0924	358	3.5.8 TCA cycle	
3630	SCF11.28C	SCO0548	361	3.6.1 Fatty acid and phosphatidic acid biosynth	
273	SC1A6.04	SCO6415	372	3.7.2 Pyrimidine ribonucleotide biosynthesis	
920	SCE8.13C	SCO3220	380	3.8.0 Secondary metabolism	3.8.0 Secondary metabolism
1025	SCE29.12C	SCO3243	380		
1050	SCF34.18	SCO0499	380		
1213	SCE29.09C	SCO3240	380		
1421	SCE29.06C	SCO3237	380		
1580	SCE29.0	SCO3233	380		
1828	SCF34.17C	SCO0498	380		
2035	SCE63.05	SCO3228	380		
2304	SCF34.09	SCO0490	380		
2566	SCE29.14C	SCO3245	380		
2765	SCE29.11C	SCO3242	380		
2963	SCE29.08C	SCO3239	380		
3113	TRPD2	SCO3212	380		
3179	SCE29.05C	SCO3236	380		
3747	SCF62.12	SCO0386	380		
4240	SCE8.14C	SCO3221	380		
4343	SCE29.13C	SCO3244	380		
4442	SCE8.11C	SCO3218	380		
4628	SCE8.08C	SCO3215	380		
83	SC3F7.03C	SCO5883	381	3.8.1 PKS	
127	REDY	SCO5880	381		
628	REDX	SCO5878	381		
774	REDD	SCO5877	381		
819	SCE29.15C	SCO3246	381		
972	SC3F7.16	SCO5896	381		
1069	SC10A5.03	SCO5898	381		
1139	REDW	SCO5879	381		
1190	SC3F7.09	SCO5889	381		
1467	SC3F7.15	SCO5895	381		
1575	SC10A5.02	SCO5897	381		
1599	SC3F7.05C	SCO5885	381		
1651	REDV	SCO5882	381		

1707	SC3F7.08	SCO5888	381		
2016	SC3F7.14	SCO5894	381		
2363	SCE29.17C	SCO3248	381		
2498	SC3F7.13	SCO5893	381		
3009	SC3F7.12	SCO5892	381		
3139	SC3F7.07C	SCO5887	381		
3361	SC3F7.04C	SCO5884	381		
4078	SC3F7.10	SCO5890	381		
4133	SCE29.16C	SCO3247	381		
4674	REDZ	SCO5881	381		
4906	SC3F7.06C	SCO5886	381		
1086	CDAR	SCO3217	382	3.8.2 NRPS	
3798	ST3F7.11	SCO5891	382		
4339	CDAPSI	SCO3230	382		
256	SCE15.06	SCO3289	416	4.1.6 Gram +ve membrane	4.0.0 Cell envelope
744	SCE15.05	SCO3288	416		
1997	SCI30A.21C	SCO1700	416		
2418	SC4G6.09C	SCO2040	416		
2551	SCC121.23C	SCO2520	416		
3003	SC9B10.01C	SCO5834	416		
3874	SCD31.18	SCO4693	416		
4221	SCE87.28C	SCO3177	416		
4844	SC5F2A.04	SCO6721	416		
4853	SCE59.12C	SCO2953	416		
684	SCC77.17C	SCO2550	417	4.1.7 Gram +ve exported/lipoprotein	
980	SCE20.11	SCO2837	417		
2278	SCM11.29C	SCO0974	417		
2933	SCF43.09	SCO0798	417		
1261	SCJ11.27C	SCO0098	514	5.1.4 Transposon/insertion element-related functions	5.0.0 Extrachromosomal
2815	SC4A2.05	SCO6369	611	6.1.1 Sensor kinase	6.0.0 Regulation
4476	SCH35.15	SCO3709	621	6.2.1 sigma factor	
4140	RSTP	SCO3943	635	6.3.5 LacI	
2620	SC5A7.31	SCO6681	641	6.4.1 Serine/threonine	
804	SCH66.08C	SCO3587	650	6.5.0 Others	
4988	SCF55.32	SCO0608	650		
100	SC9B5.09	SCO6442	700	7.0.0 Not classified (included putative assignments)	
117	SCI35.34C	SCO1612	700		
600	SC9B5.08	SCO6441	700		
1208	SC5C7.14	SCO6529	700		
1644	SC2G5.26C	SCO6205	700		
1865	SCI35.33C	SCO1611	700		
2434	SC8D9.23	SCO5511	700		
2827	SCI51.07C	SCO1767	700		
4618	SCI51.06C	SCO1766	700		
